# Adaptation of High-Altitude Plants to Plateau Abiotic Stresses: A Case Study of the Qinghai-Tibet Plateau

**DOI:** 10.3390/ijms26052292

**Published:** 2025-03-04

**Authors:** Pengcheng Sun, Ruirui Hao, Fangjing Fan, Yan Wang, Fuyuan Zhu

**Affiliations:** Southern Modern Forestry Collaborative Innovation Center, State Key Laboratory of Tree Genetics and Breeding, Key Laboratory of State Forestry and Grassland Administration on Subtropical Forest Biodiversity Conservation, College of Life Sciences, Nanjing Forestry University, Nanjing 210037, China; pcsun@njfu.edu.cn (P.S.); haoruirui99@163.com (R.H.); fanfangjing@njfu.edu.cn (F.F.); wynl@njfu.edu.cn (Y.W.)

**Keywords:** China, climatic variations, genes, metabolites, phenotype

## Abstract

High-altitude regions offer outstanding opportunities for investigating the impacts of combined abiotic stresses on plant physiological processes given their significant differences in terms of the ecological environment in high-elevation areas, low anthropogenic disturbance, and obvious distribution characteristics of plants along altitudinal gradients. Therefore, plants in high-altitude areas can be used as good targets for exploring plant adaptations to abiotic stress under extreme conditions. Plants that thrive in high-altitude environments such as the Qinghai-Tibet Plateau endure extreme abiotic stresses, including low temperatures, high UV radiation, and nutrient-poor soils. This study explores their adaptation mechanisms via phenotypic variation analyses and multiomics approaches. Key findings highlight traits such as increased photosynthetic efficiency, robust antioxidant systems, and morphological modifications tailored to high-altitude conditions. These insights advance our understanding of plant evolution in harsh environments and inform strategies to increase stress resistance in crops.

## 1. Introduction

The Qinghai-Tibet Plateau (QTP), with an average elevation exceeding 4000 m, is the world’s highest and most extensive young plateau and is renowned for its diverse and extreme terrains [1,2]. Organisms inhabiting these harsh environments undergo complex genetic adaptations, resulting in unique structural traits and sophisticated physiological mechanisms [2]. The QTP has remarkable botanical diversity, hosting over 1500 genera and 12,000 vascular plant species, accounting for more than 50% of China’s total genera and 34.3% of its vascular plant species-an ecosystem rich in plants [3]. The plateau’s geographic conditions pose significant challenges, including high altitude, low temperatures, reduced oxygen, aridity, and intense ultraviolet radiation-abiotic stressors that profoundly impact the survival and reproductive fitness of organisms [4,5,6]. Despite these adversities, resilient plant communities thrive in this demanding habitat, serving as an excellent system for examining evolutionary adaptability in high-altitude ecosystems and providing valuable insights into adaptive processes [5,7].

This review examines the intricate relationships among plant traits and the molecular mechanisms underlying their resilience to abiotic stresses in high-altitude regions of the QTP. It explores the genetic adaptations of plants to high-altitude stresses, the metabolomic responses driving their survival, and the morphological adjustments resulting from long-term adaptation to harsh environments. By synthesizing these findings, this review enhances our understanding of plant adaptation dynamics on the QTP. Additionally, this study highlights the need for further research into plant-environment interactions in high-altitude regions, offering insights into the adaptive strategies of high-altitude flora and promoting the conservation of fragile ecosystems and their ecological balance. Discussions in this field deepen our understanding of the role of abiotic stress in plant adaptive evolution while providing new perspectives for molecular and stress-resistant breeding to address global climate change. Moreover, they contribute to the preservation of fragile high-altitude ecosystems. A primary challenge in current research lies in the dynamic and complex environments of high-altitude regions, where plants face multiple abiotic stresses across varying spatial and temporal conditions. This complexity creates a critical issue: studying adaptive mechanisms to single stressors is insufficient to fully explain plant resilience under combined stresses. Many studies have revealed that plant responses to multiple stresses involve shared genes, regulatory pathways, and metabolites, as well as stress-specific mechanisms. These intricate regulatory networks pose significant challenges for deciphering plant adaptations to high-altitude environments.

**Figure 1:**
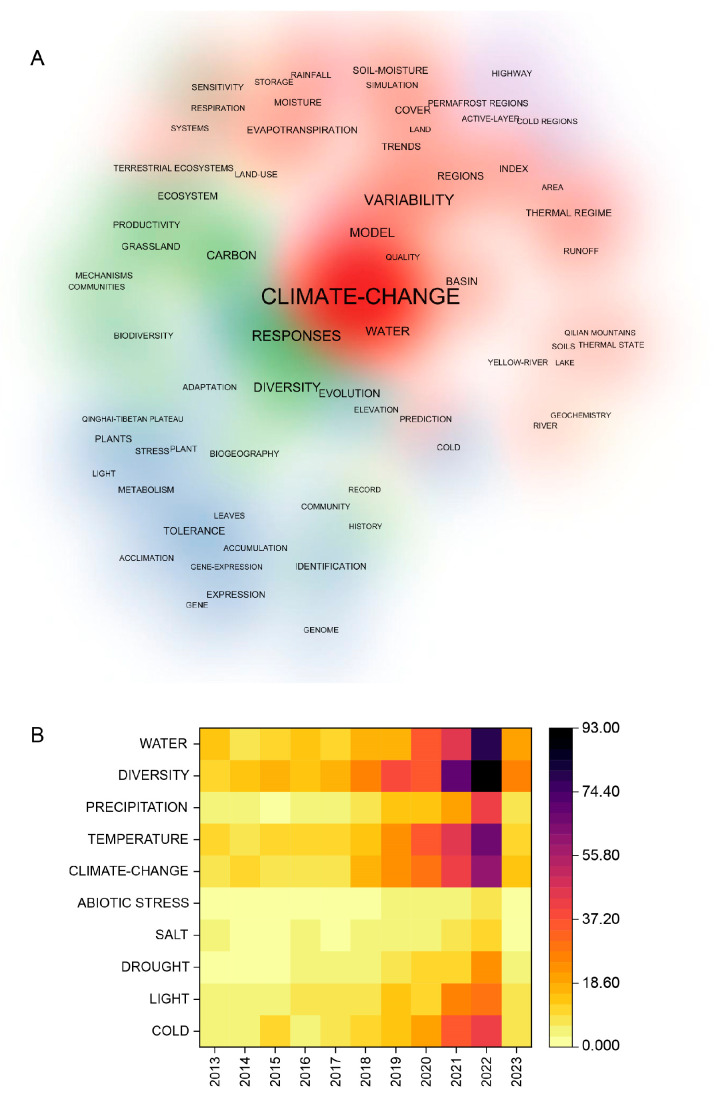
Bibliometric analysis of the response of plants to abiotic stresses on the QTP on the basis of the Web of Science core collection database. (**A**) Network diagram for bibliometric analysis of the responses of plants to abiotic stresses on the QTP. (**B**) Heatmap of bibliometric analysis, the number of documents with the keywords “Qinghai-Tibet Plateau”, “Plants”, “Abiotic stresses”, “Cold”, “High light”, “Drought”, and “Salt”.

A bibliometric study of plant responses to abiotic stressors on the QTP conducted via the Web of Science Core Collection Database demonstrated that research on plant adaptation to high-altitude abiotic stress in this region has attracted significant attention over the past decade (Figure 1). Among these studies, the adaptability of plants to different types of stress has emerged as a major research focus.

## 2. Phenotypic Changes in Plants Adapted to High Altitude

To cope with high-altitude abiotic stress, plants on the QTP undergo evolutionarily driven morphological changes, highlighting their remarkable adaptability to challenging ecological conditions. These changes are not limited to specific organs but encompass extensive structural modifications in roots, stems, leaves, flowers, fruits, and seeds. Additionally, these morphological shifts often lead to alterations in plant biomass, reflecting the multifaceted responses of plants to abiotic stressors.

### 2.1. Effects of High Altitude on Plant Root Morphogenesis

The alpine meadows of the QTP are vital resources that provide essential forage for local pastoralists [8]. However, in recent years, these ecologically sensitive meadows have experienced degradation due to the combined effects of overgrazing and rodent infestations [9]. Grazing activities significantly alter plant community composition and the intricate structure of species diversity [10,11]. Research on the effects of grazing on alpine meadow communities has focused primarily on above-ground differences between grazed and nongrazed areas, with less attention given to below-ground characteristics. Through rigorous statistical analysis of below-ground traits in both grazed and nongrazed areas, studies have revealed complex variations in the root traits of high-altitude plants, providing critical insights into their adaptive strategies.

**Figure 2:**
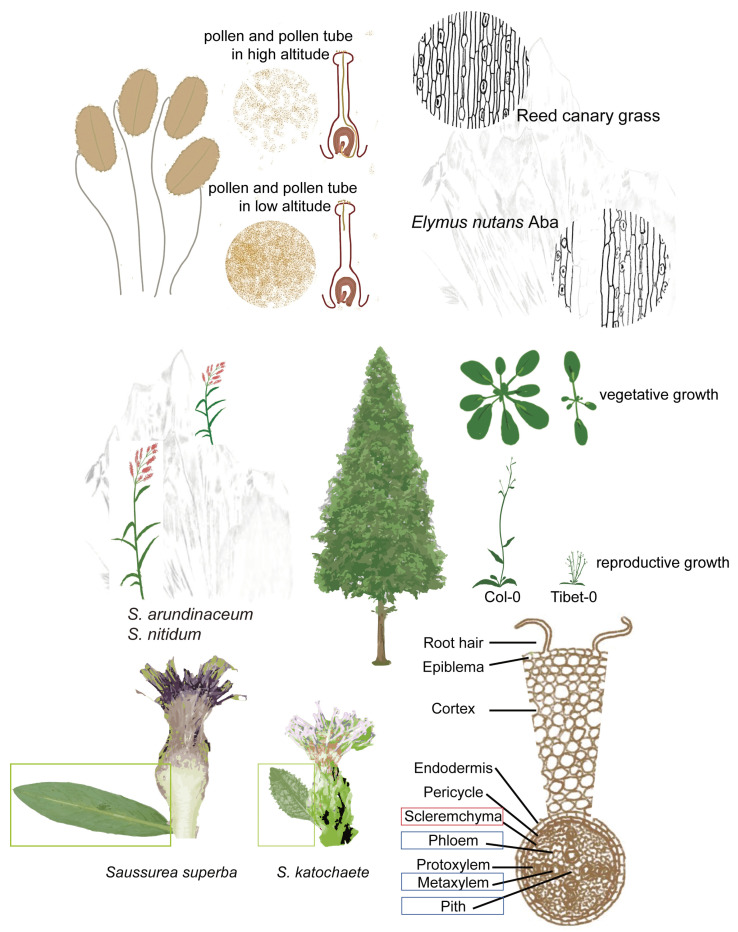
Morphological changes in plants in response to abiotic stresses on the QTP. The morphological changes in the plants distributed on the QTP are summarized. The roots, plant heights, stomata, pollen, leaves, and leaf angles were analyzed. The indicators delineated by red boxes correspond to root parameters exhibiting a positive correlation with elevational gradient, whereas those demarcated by blue boxes represent root variables demonstrating an inverse relationship with increasing altitude. The green box highlights the leaf morphological differences between *S. superba* and *S. katochaete*.

In the harsh environment of the plateau, plants on the QTP generally grow slowly, and research on Aveneae Dumort species has shown that as elevation increases, there are diverse responses to plateau stress. Some grass species show increased growth, whereas others decline. The thickness of sclerenchyma tissues uniformly increases with elevation across all the grass species, with *Polypogon monspeliensis* exhibiting maximal sclerification at relatively high altitude. However, traits related to the stele (such as the size of the metaxylem region, the extent of the phloem region, the width of the pith, and the area of pith cells) decrease with increasing elevation [12] (Figure 2). Notably, sclerification, particularly within the vascular region, is the primary response of all grass species to increasing altitude, although the degree of sclerification varies significantly among species [12].

Studies on *Sorghum* species have revealed the morphological characteristics of *Sorghum* at different elevations. Analysis of the growth environment and plant traits of two *Sorghum* species (*Sorghum nitidum* and *Sorghum arundinaceum*) revealed that the root length and root radius of *S. nitidum* increase with elevation, accompanied by a complete breakdown of the dermal and cortical zones and a significant increase in endodermal thickness. In contrast, *S. arundinaceum* maintains a constant root length, while its root radius decreases with elevation. The area of cortical cells in *S. arundinaceum* steadily decreases with elevation, whereas in *S. nitidum*, the cortical cell area decreases only at the highest elevation. The thickness of both *S. nitidum* and *S. arundinaceum* significantly increases with elevation, but the thickness of the pericycle and the area of metaxylem vessels decrease in both species, particularly in *S. arundinaceum.* The metaxylem area of *S. arundinaceum* remains constant with elevation, whereas the phloem thickness decreases significantly. The phloem thickness of *S. nitidum* decreases even more markedly. Additionally, the pith thickness of *S. arundinaceum* remains unchanged with elevation, whereas that of *S. nitidum* continues to decrease as elevation increases [13].

### 2.2. Effects of Altitude on Stem Morphological Changes

The stem heights of both *Sorghum* species (*S. arundinaceum* and *S. nitidum*) decreased with increasing altitude (Figure 2). In *S. arundinaceum*, the cellular region reached its maximum thickness at 1800 m, whereas in *S. nitidum*, the cellular region gradually thinned with increasing elevation. Additionally, in *S. arundinaceum*, all stem features-including the dermal layer (epidermal thickness), fundamental tissue (cortical parenchyma and supporting tissue), and vascular system-decrease as altitude increases [13].

The morphology of plant stems on the Tibetan Plateau was also influenced by UV-B radiation. A study investigated the effects of enhanced UV-B radiation on the growth, morphology, reproduction, and biophysical characteristics of three *Fagopyrum esculentum* Moench (buckwheat) cultivars originating from both high-altitude regions (Qinghai-Tibet Plateau) and low-altitude areas (Sichuan Basin). The stem diameter of all three cultivars from high-altitude regions decreased with increasing UV-B levels [14].

### 2.3. Effect of Altitude on Plant Leaf Morphology

On the QTP, alterations in leaf traits are more pronounced than those in other plant organs. As the primary organ for photosynthesis and water regulation, leaves are significantly affected by the intense sunlight, aridity, and low temperatures of the plateau. Consequently, vegetation in this region undergoes morphological changes in leaves as an adaptation to these stressors.

Previous sections have detailed the root and stem structural changes in *S. arundi-naceum* and *S. nitidum* [13]. Notably, the leaf characteristics of these two species also exhibit distinct variations. In *S. arundinaceum*, the midrib thickness increases significantly with elevation, whereas the blade thickness decreases. Conversely, in *S. nitidum*, the midrib thickness decreases, whereas the blade thickness increases with altitude. Interestingly, trichomes are present in *S. arundinaceum* and proliferate with increasing elevation. Both species presented notable decreases in stomatal density at mid-altitudes. However, *S. arundinaceum* has the highest stomatal density at the highest altitude, whereas *S. nitidum* has the highest density at the lowest altitude [13].

Stomatal density is a critical indicator for studying leaf traits in plateau plants. A comparative study of reed canary grass (a persistent energy-yielding grass) and *Elymus nutans* Aba (a native perennial grass) revealed that the stomatal density of reed canary grass is significantly greater than that of *E. nutans* Aba, while its stomatal pore size is smaller (Figure 2). These findings suggest a potential correlation between stomatal density, pore size, and cold resistance [15].

A study of *Stipa purpurea*, which is known for its enhanced drought resistance, revealed that the leaf surface of *S. purpurea* contains more cuticular wax crystals than does that of *Stipa capillacea*. These findings indicate a potential link between cuticular wax and drought resistance. Additionally, cuticular wax crystals are more abundant on *S. purpurea* leaves than on *S. capillacea leaves*. The water loss rate of *S. purpurea* is significantly lower than that of *S. capillacea*, suggesting that cuticular wax plays a role in the drought protection mechanism of *S. purpurea* [16].

Morphological differences are evident in the leaves of *Saussurea superba* (prostrate) and *Saussurea katochaete* (erect) from high elevations of the QTP. *S. superba* leaves grow horizontally, whereas *S. katochaete* leaves grow upright (Figure 2). Compared with that of *S. katochaete*, the horizontal growth of *S. superba* results in a greater photosynthetic photon flux density (PPFD). Additionally, the leaf temperature of *S. superba* was greater than that of *S. katochaete*. Plant structure influences light absorption and leaf temperature, leading to varying effects on photoinhibition. Research indicates that upright leaves are more sensitive to high temperatures, whereas horizontal leaves can withstand greater PPFD. These findings suggest that intense radiation on plateaus may regulate the balance between light energy absorption and potential light damage in horizontal leaves through rapid and reversible photoinhibition [6].

*Arabidopsis thaliana*, a widely used model plant, exhibits morphological adaptations on the QTP. Compared with the Col-0 ecotype of *A. thaliana*, the Tibet-0 ecotype (from the plateau) has fewer leaves during the vegetative growth stage, more branches during the reproductive stage, and a shorter plant height, reflecting typical plateau plant characteristics [17] (Figure 2). Further studies revealed that, compared with Col-0, Tibet-0 has greater resistance to high light stress. While their chlorophyll contents are similar, Tibet-0 plants accumulate significantly fewer anthocyanins under high light stress, indicating reduced sensitivity to intense light [18].

In the chloroplasts of both Col-0 and Tibet-0, relocation from the cell surface to the cell side was observed, with the light aligning with the direction of incident light to reduce absorption. An examination of the chloroplast structure revealed that the stacked grana thylakoids and unstacked stroma thylakoids in Tibet-0 were markedly distended, with an enlarged stromal void. In contrast, no significant swelling was observed in the thylakoid membrane system of Col-0. Thus, chloroplast morphological changes may contribute to Tibet-0′s adaptation to high-intensity light stress [18].

Morphological adaptations in herbaceous plants on the plateau are conspicuous, featuring traits such as short stature and creeping leaves. However, certain woody plants also exhibit structural modifications in response to harsh environments. Previous studies have indicated that lower temperatures at higher elevations restrict leaf expansion and reduce leaf size [19]. High-altitude solar radiation contributes to reduced dry matter, a smaller overall size, and shorter leaf petioles [20,21]. *Quercus guyavifolia* H. Léveillé (Fagaceae), a woody plant native to the southeastern boundary of the plateau, is distributed from 2000 m to 4500 m, providing an excellent opportunity to study the relationships between leaf traits and ecological factors. Research has shown significant decreases in the leaf area, length, width, length-width ratio, form factor, petiole length, and width of *Q. guyavifolia* with increasing altitude. These seven traits are positively correlated with the mean annual precipitation. However, the leaf length, length-to-width ratio, and petiole length were negatively correlated with the maximum daily average UV-B irradiance during the growth period. These marked changes associated with elevation may play a crucial role in the effective acclimation of *Q. guyavifolia* along the plateau boundary [22].

In general, high-altitude leaves adapt to abiotic stress through morphological changes. Plants in these regions, including the QTP, typically exhibit stunted growth, smaller and rounder leaves with enhanced trichomes, and curled leaves that form concealed chambers. These adaptations distinguish them from their lowland counterparts [23].

These plants often display high leaf and branch densities or maintain persistent leaves in rosette formations. Additionally, some species exhibit unique morphologies, such as “greenhouse” plants with specialized floral bracts or foliage and “woolly” plants covered with long, thick hairs that function as heat accumulators, accelerating development and reproduction. These adaptations are crucial in the plateau’s inhospitable conditions, where the growing season is brief (typically 3–4 months) and vegetation is sparse [24].

### 2.4. Floral and Seed Changes in Plants

The harsh natural environment of the QTP also poses challenges for plant reproduction. During flowering, plants in this region are subjected to UV-B stress, resulting in a decrease in the number of anthers [14].

Additionally, studies have explored pollen germination and pollen tube development in species from different areas of the plateau. Although some plants present reduced pollen quantity [14], their pollen germination and pollen tube growth are greater than those at lower elevations. These findings suggest that certain species on the plateau have greater pollen tolerance than their low-altitude counterparts [25] (Figure 2).

Seed morphology on the QTP is also influenced by abiotic stress, and changes in seed morphology increase germination rates. Generally, both seed shape and size influence germination rates, as does seed quantity. Compared with compact seeds, elongated seeds presented greater germination percentages and faster germination rates. In contrast, smaller seeds have greater germination potential than larger seeds do. Seed volume and shape evolve in tandem but exert independent effects on germination. Specifically, seed volume does not significantly affect the germination of species with compact seeds but has a notable effect on those with elongated seeds. For larger-seeded species, germination is influenced more by seed shape than by seed volume [26].

## 3. Gene Regulation in Response to Abiotic Stress in Plants

The unique geographical environment of the QTP has shaped a distinctive ecological landscape. The diversity of biological species provides a rich genetic basis for plant resistance to abiotic stress [27], enhancing our understanding of the molecular mechanisms underlying plant adaptation to such stress in high-altitude regions [28].

Studies on the effects of low temperature on plant maturation and development in high-altitude regions have identified numerous genes that regulate plant adaptation to low-temperature stress [29,30,31].

### 3.1. Genes Involved in Cold Stress Regulation in Plants

Low temperature induces changes in plant morphological structure and gene expression [32]. Under low temperature, biochemical reaction rates in plants decrease, membrane fluidity and protein conformation change, enzyme activity decreases, and even water crystallization can damage plant cells [29,33].

**Figure 3:**
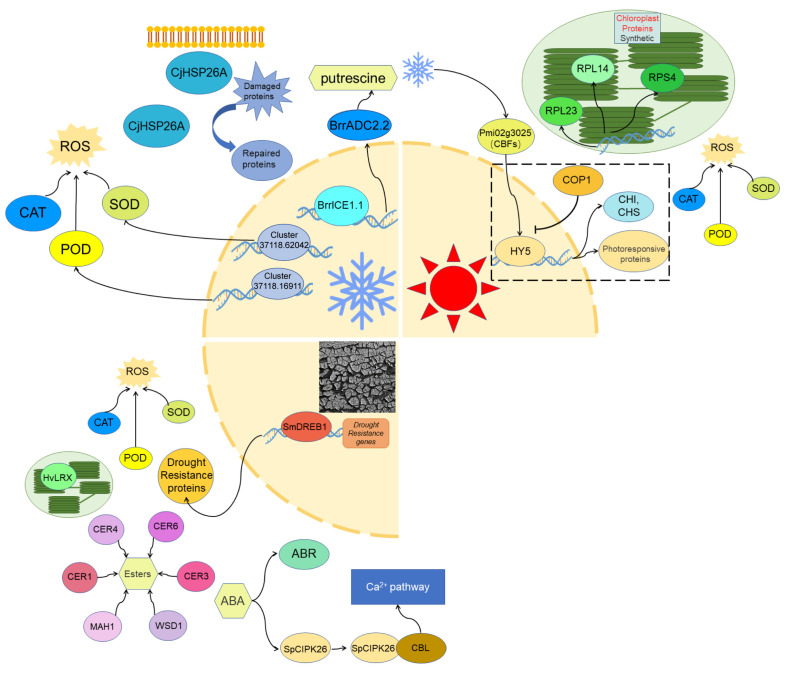
Gene regulatory pathways in plant responses to various abiotic stresses. Molecular mechanisms of plant responses to abiotic stresses such as cold, high light and drought stress, including genes and signaling pathways, studied in the plants of QTP. Circles and ovals represent proteins, hexagons represent metabolites, and the yellow three-quarter circle represents the nucleus. Abbreviations: BrrADC2.2, *Brassica rapa* Related Arginine Decarboxylase 2.2; BrrICE1.1, *Brassica rapa* Related Inducer of CBF Expression 1.1; CAT, Catalase; CBL, Calcineurin B-Like Protein; CER, Cytokinin Response; CHI, Chalcone Isomerase; CHS, Chalcone Synthase; CjHSP26A, *Cinnamomum japonicum* Heat Shock Protein 26A; COP1, Constitutive Photomorphogenic 1; HvLRX, *H. vulgare* Leucine-Rich Repeat Extension; HY5, Elongated Hypocotyl 5; MAH1, Multicopper Oxidase-Like Protein 1; POD, Peroxidase; ROS, Reactive Oxygen Species; RPL, Ribosomal Protein Large subunit; SmDREB1, *Sorghum bicolor* Dehydration Responsive Element Binding Protein 1; SOD, Superoxide Dismutase; SpCIPK26, *Solanum pennellii* Calcium-Dependent Protein Kinase 26; WSD1, Wax Synthase/Diacylglycerol Acyltransferase 1.

Numerous genes have been shown to participate in plant responses to low-temperature stress. Additionally, most genes associated with the reactive oxygen species (ROS) signaling pathway are involved in plant stress response signaling [34]. These genes also play a role in the response of plants to low-temperature stress in high-altitude regions of the QTP [35] (Figure 3).

The cold stress response mechanisms in plants from the QTP are similar to those in other regions [31]. Under cold stress, the ROS system of plants on plateaus is activated [36]. Superoxide stress induced by cold stress triggers the expression of oxidative stress-related genes, such as *SOD*, *POD*, *APX*, and *CAT* [35,37,38]. In *Helictotrichon virescens*, *Cluster37118.16911* acts as an upstream regulator of POD activity, whereas *Cluster37118.62042* regulates SOD activity [30]. With the assistance of *SOD*, *POD*, *APX*, and *CAT*, plants can effectively mitigate superoxide stress, eliminate oxidative free radicals, prevent excessive oxidation of the plasma membrane, and protect themselves from cold stress [30,31,39] (Figure 3).

The low-temperature stress response protein LEA is also involved in the cold stress response of plants on the QTP [40]. LEA proteins are widely implicated in plant responses to cold stress [41]. *Caragana jubata* on the plateau respond to cold stress through genes such as *CjLEA-Dc3*, *CjLEA-CI*, and *CjLEA-14*. Additionally, the *CjHSP26A* gene in this plant protects the cell membrane structure, refolds denatured proteins, and prevents protein aggregation, thereby safeguarding the plant under low-temperature conditions [40].

In addition to functional proteins, many regulatory factors are involved in the cold stress response of plateau plants [42]. In a study on *Helictotrichon virescens*, numerous transcription factors were shown to participate in the plant response to low-temperature stress. These include *AP2/ERF*, *FAR1*, *TRAF*, *C2H2*, *bHLH*, *B3*, *bZIP/GRAS*, *C3H*, *AUX/IAA,* and *GARP-G2-like* transcription factors [30]. These transcription factors are also widely reported to be involved in cold stress resistance pathways in low-altitude plants [43]. Although research on the transcription factors involved in plant adaptation to high-altitude cold stress on the QTP is limited, some progress has been made. The *Brassica rapa* var. *rapa* transcription factor *BrrICE1.1*, a homolog of *ICE1*, directly binds to the *BrrADC2.2* promoter, activating *BrrADC2.2* to promote putrescine accumulation and increase cold stress resistance [42] (Figure 3).

### 3.2. Genes Related to the Plant Response to High Light and Ultraviolet Stress

The QTP is characterized by intense light and ultraviolet (UV) radiation [44], which can severely affect plant photosynthesis [45]. Strong light and UV rays can damage chloroplasts, and UV radiation can also induce the accumulation of superoxide anions, leading to oxidative stress in plants [30,46]. Plants on plateaus have developed strategies to cope with high light and UV stress [47]. Consequently, genes such as *SOD*, *POD*, *CAT*, and *APX*, which respond to oxidative stress, are also involved in plant adaptation to high light stress [14,47] (Figure 3).

In addition to the ROS response mechanism, other genes, particularly those encoding chloroplast proteins, play a role in plant responses to high light and UV stress on plateaus [48,49]. In *A. thaliana*, the expression levels of the chloroplast ribosomal proteins S4 (RPS4), L14 (RPL14), and L23 (RPL23) in the Tibet-0 ecotype were greater than those in the Col-0 ecotype. After high-light treatment, the expression of these chloroplast ribosomal proteins further increased in Tibet-0 [18] (Figure 3). Chloroplast ribosomal proteins are essential for chloroplast protein synthesis, indicating that Tibet-0 maintains a high level of chloroplast translation capacity under high light stress, enabling rapid synthesis of photosynthetic proteins [18,50] (Figure 3).

In addition to the use of ROS pathways and chloroplast protein protection, plants on the QTP employ additional strategies to cope with high light stress [18,47]. A study on *Prunus mira* seedlings subjected to low temperature and UV-B radiation revealed significant upregulation of C-repeat binding transcription factor (CBF) and late embryogenesis abundant protein (LEA) expression under cold induction. Additionally, the expression of thaumatin-like proteins increased in response to UV-B induction. The CBF gene *Pmi02g3025* in *P. mira* was upregulated under both chilling and UV stress [51]. While the role of CBF transcription factors in cold stress has been previously discussed [52], their involvement in UV stress in high-altitude plants remains unclear. However, other studies provide insights into how CBF may respond to high light stress in such plants [53].

Initial investigations in *A. thaliana* revealed that light is essential for cold stress tolerance. HY5, a key regulatory element in photomorphogenesis controlled by the E3 ubiquitin ligase COP1, was identified in these studies [54]. COP1 is a crucial inhibitor of light signal transduction [55]. Under light conditions, COP1 is removed from the nucleus, where it stabilizes HY5 and activates light-responsive genes [55]. Approximately 10% of cold-induced genes related to cold acclimation in *Arabidopsis* are regulated by the HY5 pathway, which includes HY5, COP1, Z-box elements, and motifs involved in photoregulatory gene expression [56]. HY5 transcription is regulated by low temperature through CBF- and ABA-independent pathways [53]. Under cold stress, HY5 induces the expression of cold-induced genes, including CHS (chalcone synthase) and CHI (chalcone isomerase) [57] (Figure 3). Anthocyanin accumulation protects plant photosystems from ROS accumulation during abiotic stress [58].

### 3.3. Genes Involved in Drought Regulation in Plants

Despite the predominance of low-temperature and high-light stresses on the QTP due to its high altitude, the region also experiences significant variability in precipitation owing to its unique geographical environment [59,60]. Although the Yarlung Zangbo River area has a rich water system, the Tibetan Plateau’s high-altitude terrain restricts the southwest monsoon from delivering sufficient water vapor, resulting in widespread aridity [61]. Consequently, plants in this region have evolved molecular mechanisms to adapt to drought stress [7].

*Sophora moorcroftiana*, a plant native to the Tibetan Plateau, exhibits remarkable drought tolerance [62]. Transcriptome sequencing has revealed numerous genes associated with drought stress responses, including those encoding transcription factors from the AP2/ERF, NAC, MYB, Zn-Finger, and WRKY superfamilies [62]. These transcription factors, particularly AP2, NAC, MYB, Zn-Finger, and WRKY, are known to respond to drought stress in various species [63,64] (Figure 3). Additionally, far-red response 1 (FAR1) has been classified as a drought-responsive gene [65], indicating that high-altitude plant responses to drought are conserved across species.

Other drought-related genes have also been reported. For example, the ABA-responsive (ABR) gene, associated with ABA signaling, is induced under drought conditions, whereas the auxin-induced protein (AIP) linked to IAA is downregulated. These findings suggest that the drought resistance of *S. moorcroftiana* is mediated through a hormone-regulated signaling network [62] (Figure 3).

Further research on high-altitude drought stress responses identified *SpCIPK26* in *S. purpurea*. Notably, this gene also confers salt stress tolerance. The heterologous expression of *SpCIPK26* in *Arabidopsis* enhances ABA-dependent tolerance to both drought and salt stress [66].

Genes associated with other abiotic stresses also play roles in drought responses. For example, mechanisms enhancing chloroplast ribosome synthesis to resist high-light stress [18] may also be relevant. Under drought stress, the expression of ribonucleoproteins (*SPOT3s*), which are involved in protein translation and synthesis, increases, suggesting that high-altitude plants employ strategies similar to those of other species to cope with drought [62].

Similar to how plants respond to cold and light stress, high-altitude plants mitigate drought stress by reducing reactive oxygen species (ROS) [67]. While common ROS-scavenging genes such as *SOD*, *POD*, *CAT*, and *APX* are not discussed in detail, *S. moorcroftiana* exhibits increased expression of *GPDH* under drought stress, a gene with homologous responses in *A. thaliana* [62] (Figure 3).

Drought also influences the expression of *CHI* and *CHS* genes [62]. These genes, which are regulated by HY5, a gene associated with cold stress and photomorphogenesis, suggest that *HY5* may also mediate drought responses in high-altitude plants [57].

To combat drought, plants increase water absorption and minimize water loss. Natural selection favors dehydration avoidance over tolerance [16,68,69]. For example, *Stipa* species synthesize waxes to prevent water loss, with increased expression of wax synthesis-related enzymes under drought stress [16]. Wax synthesis occurs in three stages: (1) fatty acid synthesis [70]; (2) elongation by a complex including β-ketoacyl-CoA synthase, reductase, dehydratase, and enoyl-CoA reductase; and (3) final synthesis involving fatty acyl-CoA reductases (*CER1*, *CER3*, *CER4*) and wax ester synthase/diacylglycerol acyltransferase (*WSD*) [71,72] (Figure 3).

In *S. capillacea* and *S. purpurea*, *CER1* expression increased 7.5-fold and 14-fold, respectively, after drought treatment. *CER1*, *CER3*, *CER4*, and *CER6* expression levels were significantly higher in *S. purpurea* than in *S. capillacea*, whereas *WSD1* and *MAH1* showed the opposite trend. These findings suggest that *CER1*, *CER3*, *CER4*, *CER6*, *WSD1*, and *MAH1* regulate wax synthesis to increase drought resistance in high-altitude plants [16].

RNA-seq analysis of *S. moorcroftiana* revealed numerous drought resistance genes, including those encoding transcription factors, plant hormone regulators, cell structure stabilizers, oxidative stress genes, and secondary metabolic genes [62]. Among these genes, *SmDREB1* was found to upregulate drought resistance genes in *Arabidopsis*; increase SOD, POD, and CAT activities; and improve photosynthetic efficiency [5]. *DREB* genes, which were previously linked to chilling stress, appear to be central to high-altitude plant adaptation to multiple abiotic stresses [5] (Figure 3).

In Tibetan hulless barley (*Hordeum vulgare* L. var. *nudum* hook. F.), *HVU010048.2* (*HvLRX*), a chloroplast-localized drought tolerance gene, was identified. This gene is induced under high light and repressed in darkness, with its promoter region containing light-responsive elements such as the ATC motif, Box 4, G-box, Sp1, and chs-CMA1a. *HvLRX* responds to high light, dehydration, progressive drought, PEG 6000, and NaCl treatments but is insensitive to ABA. This gene is the first gene in the QTP to respond simultaneously to both drought stress and photosignal stress [73].

### 3.4. Genes Involved in Salt Stress Regulation in Plants

The QTP is home to extensive grasslands, which have faced increasing soil desertification in recent years due to overgrazing and other factors, resulting in severe salt-alkali stress to plants [74]. Like the dual role of *SpCIPK26* in *S. purpurea*, which responds to both drought and salt stress [66] (Figure 3), plants on the Tibetan Plateau have developed molecular mechanisms to cope with salt stress.

Previously, we discussed the role of *CIPK26* in drought stress responses [66]. In fact, *CIPK* interacts with calcineurin B-like proteins (*CBLs*) to regulate numerous biological processes through the Ca^2+^ signaling pathway [75], a mechanism conserved across many species [76,77,78]. The *CBL-CIPK* module also underpins signaling pathways for plant resistance to salt stress [79], cold stress [80], drought stress [66], and ABA responses [81].

## 4. Metabolites of Plants Responding to Abiotic Stress on the Tibetan Plateau

Numerous genes are involved in the acclimation of plants to abiotic stress in high-altitude regions of the QTP. Simultaneously, plants in this region produce a wide array of metabolites that play crucial roles in their adaptation to such stresses. These genes include transcription factors, which regulate downstream gene expression and modulate plant responses to abiotic stress, as well as functional genes that facilitate stress tolerance through the production of primary and secondary metabolites such as hormones and waxes. Both primary and secondary metabolites significantly contribute to plant responses to abiotic stress on the QTP.

### 4.1. Role of Primary Metabolites in Plant Responses to Abiotic Stress

Primary metabolites are essential for plant responses to abiotic stress on the QTP. These metabolites include soluble proteins, soluble sugars, and proline. Studies have shown that the levels of these compounds increase with altitude, aiding plants in adapting to harsh environmental conditions [31].

Soluble proteins, the most abundant macromolecules in plants, are vital for sustaining life activities [82]. Their concentration influences osmotic regulation within plant cells. Higher protein levels enable cells to maintain a low osmotic potential, enhancing water absorption and storage capacity. This phenomenon supports cell growth and improves stress tolerance [83,84].

Plants often adapt to osmotic stresses, such as the low temperatures typical of high-altitude regions, by increasing the levels of osmotic regulators such as proline and total soluble sugars (TSSs). These compounds play critical roles in mitigating environmental challenges [31,85]. Soluble sugars, the primary osmotic regulators in plants, reduce the damaging effects of low temperatures. Under cryogenic stress, elevated soluble sugar concentrations increase the cell sap concentration, lowering the freezing point, preventing excessive dehydration, and inhibiting protoplast coagulation, thus enabling adaptation to cold stress [86]. Proline is particularly important for plants on the QTP, as a high proline content helps alleviate drought stress [87], cold stress [31], and high-light stress [51].

### 4.2. Role of Secondary Metabolites in Plant Responses to Abiotic Stress

Phytohormones are crucial in plant responses to abiotic stress. Key hormones such as ABA, auxins, gibberellins (GA), cytokinins (CTK), ethylene (ETH), and brassinosteroids (BR) are involved in adaptation to cold, salt, and drought stress [88]. ABA, a central hormone in stress responses, regulates plant water status, enhances osmotic stress tolerance, and facilitates adaptation to abiotic stress [88,89]. On the QTP, ABA levels increase significantly with altitude, indicating its role in high-altitude adaptation [31,90]. For example, *SpCIPK26* gene in *S. purpurea* mediates drought and salt stress tolerance through ABA-related signaling pathways [66]. However, research on the role of ABA and other hormones in high-altitude adaptation remains limited. Auxin levels decrease with altitude [31], whereas studies on GA, CTK, ETH, and BR are primarily based on omics data, with limited exploration of their regulatory roles [91,92]. Interestingly, chlorophyll, a vital photosynthetic pigment, also participates in plant responses to abiotic stress at high altitudes [93].

High-altitude plants reduce chlorophyll expression through gene regulation, minimizing light absorption and mitigating high-light stress [94]. In *Quercus aquifolioides*, the chlorophyll content initially increases with altitude due to light induction but decreases at relatively high elevations due to UV-B radiation and relatively low temperatures [95]. Chlorophyll also acts as a stress-resistant metabolite. For example, cold-tolerant *E nutans* varieties present higher chlorophyll levels than do less cold-tolerant varieties [96]. Elevated chlorophyll supports photosynthesis, enhances organic matter accumulation, reduces osmotic potential, and improves cold tolerance [97].

Other metabolites also play key roles in plant adaptation to high-altitude stress. Malondialdehyde (MDA), a product of lipid peroxidation, damages cell membranes [98]. High-altitude plants present lower MDA levels than low-altitude plants do, as altitude-induced stress activates ROS systems [99,100,101]. Anthocyanins accumulate with decreasing temperature, protecting plants from cold stress [102]. Plant waxes, which are synthesized to combat drought stress, also contribute to high-altitude adaptation. For example, *S. purpurea* presents higher levels of C24-C32 primary alcohols, alkanes, and C38-C40 esters than *S. capillacea does*, with further increases under drought stress [16].

Flavonoids, multifunctional secondary metabolites, protect plants from abiotic stress [103]. Drought-induced osmotic stress increases ROS, causing cell damage or death [104]. High flavonoid levels in Tibetan Plateau plants are linked to stress adaptation [94,105,106]. Additionally, high melatonin (MEL) levels in *H. vulgare* L. contribute to salt stress tolerance [107,108].

Other metabolites, such as pyridoxine O-glucoside, L-alanine, kynurenic acid, 2′-deoxyadenosine-5′-monophosphate, and nicotinate ribonucleoside, respond to salt stress [106]. Metabolic pathways such as starch and sucrose metabolism, the pentose phosphate cycle, flavonoid biosynthesis, phenylpropanoid biosynthesis, the citric acid cycle, and amino acid metabolism are activated under cold stress [109].

Compared with primary metabolites, secondary metabolites, including plant hormones, chlorophyll, anthocyanins, flavonoids, and plant waxes, play a more prominent role in plant defense against abiotic stress on the QTP. These metabolites are essential for understanding plant responses to abiotic stress at high altitudes. Stress-resistant metabolites reveal adaptation mechanisms, whereas marker metabolites help assess stress severity and guide protective measures.

## 5. Discussion and Future Perspectives

In summary, the adaptability of plants on the QTP to high-altitude abiotic stress is increased through changes in genes and metabolites. Over evolutionary timescales, high-altitude plants have developed biological traits and morphological adaptations to cope with the unique stresses of their environment.

Currently, the molecular mechanisms underlying plant responses to abiotic stress in the Qinghai-Tibet region are not fully understood, but in-depth research remains limited. Most studies rely on omics data, yet the analysis and mining of these datasets are insufficient. Additionally, many candidate genes identified through omics approaches await further investigation, offering rich opportunities for future research.

The molecular mechanisms of common abiotic stresses on the Tibetan Plateau-such as cold, high light and UV, drought, and salt stresses-have been well studied at relatively low altitudes. For example, transcription factors such as DREB1 and CBF, which contain AP2 elements, are critical for cold stress responses. *CBF* is rapidly induced at low temperatures, activating the expression of *COR* genes and promoting the accumulation of cryoprotective proteins [52,110]. The *ICE1* gene, which contains a bHLH element, induces *CBF* expression [111]. Under cold stress, the myristoylation of protein phosphatases reduces EGR2 function, decreasing its interaction with OST1 (OPEN STOMATA 1), which increases kinase activity. OST1 phosphorylates ICE1, increasing its transcriptional activity and stabilizing it by reducing its binding to E3 ubiquitin ligases [43,111,112].

Insights from low-altitude plants may be applicable to high-altitude species, although unique regulatory mechanisms in the QTP require further exploration. Future research should focus on identifying novel signaling pathways and understanding phenomena such as single genes (e.g., CIPK, CBF) that participate in multiple stress responses.

Alternative splicing plays a significant role in modulating plant stress responses. It has been shown to be essential for cold [113], salt [114,115], and drought [116] stress responses. Investigating whether high-altitude plants possess unique alternative splicing mechanisms to cope with abiotic stress is an intriguing area for future studies.

In conclusion, plants on the QTP provide valuable material for exploring plant resistance to abiotic stress. Their adaptations to harsh environments offer a theoretical foundation for breeding superior species with tolerance to cold, high light, drought, and poor soil conditions.
